# Dynamics of Cell Ensembles on Adhesive Micropatterns: Bridging the Gap between Single Cell Spreading and Collective Cell Migration

**DOI:** 10.1371/journal.pcbi.1004863

**Published:** 2016-04-07

**Authors:** Philipp J. Albert, Ulrich S. Schwarz

**Affiliations:** Institute for Theoretical Physics and BioQuant, Heidelberg University, Heidelberg, Germany; Johns Hopkins University, UNITED STATES

## Abstract

The collective dynamics of multicellular systems arise from the interplay of a few fundamental elements: growth, division and apoptosis of single cells; their mechanical and adhesive interactions with neighboring cells and the extracellular matrix; and the tendency of polarized cells to move. Micropatterned substrates are increasingly used to dissect the relative roles of these fundamental processes and to control the resulting dynamics. Here we show that a unifying computational framework based on the cellular Potts model can describe the experimentally observed cell dynamics over all relevant length scales. For single cells, the model correctly predicts the statistical distribution of the orientation of the cell division axis as well as the final organisation of the two daughters on a large range of micropatterns, including those situations in which a stable configuration is not achieved and rotation ensues. Large ensembles migrating in heterogeneous environments form non-adhesive regions of inward-curved arcs like in epithelial bridge formation. Collective migration leads to swirl formation with variations in cell area as observed experimentally. In each case, we also use our model to predict cell dynamics on patterns that have not been studied before.

## Author Summary

The collective dynamics of many cells is more than the sum of its parts. For example, large cell collectives often form streams, swirls or bridges that cannot be achieved by single cells. Yet the dynamic processes of single cells, especially their response to adhesive and mechanical cues, stays an essential element of the collective cell dynamics. Here we introduce a comprehensive modeling framework that allows us to predict cellular dynamics from the level of single cells up to the level of large cell collectives on the same footing. We focus on cellular dynamics on adhesive micropatterns as an especially successful approach to investigate and control complex cell behaviour. Our model successfully predicts a large range of experimentally observed phenomena, allows us to investigate the relative importance of the different cellular processes and in the future can be used to design new adhesive micropatterns that promote desired cell dynamics.

## Introduction

Adhesive micropatterns (MP) determine the spatial distribution of the extracellular matrix (ECM) and therefore allow us to investigate and control cell shape, structure and function through experimental design. Over the last decade, they have emerged as an extremely versatile tool to investigate the inner workings of cells [1]. In particular, they are especially suited to achieve a quantitative understanding of how cells respond to external cues. Pioneering work with adhesive micropatterns has demonstrated the importance of the ECM-geometry for the survival of cells [2]. Later work showed how e.g. the organisation of the cytoskeleton [3, 4] and of endomembranes [5] depend on ECM-geometry. Adhesive micropatterns have also been used to address the mechanical aspects of cells [6, 7]. The versatility of adhesive micropatterns is further increased by combination with traction force microscopy on soft elastic substrates [8–10]. Although originally designed to immobilize single cells, micropatterns have also been extensively used to study their dynamic processes, including the different phases of cell spreading [3] or migration on stripe patterns with a focus on cell speed and persistency [11, 12].

During recent years, the micropatterning approach has been increasingly applied also to multicellular systems. A first step towards multicellular systems is division of single cells which has been investigated with a focus on the central question how the cell division axis is determined by ECM-geometry [13, 14]. It has been found that the statistical distribution of the direction of the cell division axis has a clear relation to the ECM-geometry. It has been argued that this relation is mainly provided by so-called retraction fibers that anchor the dividing cell to the adhesive micropattern [14, 15]. The result of a division are usually two daughter cells that share one micropattern. Already this simple situation leads to very rich behavior. Cell pairs on square or circular micropatterns usually undergo a spontaneous symmetry break, assume a Yin-Yang shape and rotate persistently in the direction of the blunt cell sides [16]. Occasionally rotation can stop and cells rearrange, but eventually the cells resume rotation, either in the same or the opposite direction. Going beyond square or circular patterns, it has been found that the geometrical details of the pattern strongly influence the rotational behavior. In particular, it can be suppressed by using concave patterns that force the cells to deviate substantially from a circular trajectory [17].

Micropatterns are also increasingly used to study collective cell migration of large ensembles, mainly of monolayers of epithelial cells like MDCK-cells [18]. Collective cell migration is a hallmark of large scale cellular rearrangements during development, wound healing and cancer invasion [19–21]. Although cells moving collectively as sheets, strands, streams, clusters or tubes are often observed in three-dimensional situations, the two-dimensional setup with monolayers allows a more detailed analysis and in particular the use of micropatterns. The classical scratch-assay for two-dimensional wound healing assays has been replaced early by a microfabrication approach in which removable barriers allow to initiate collective cell migration into the gap without causing debris and ill-defined borders [22]. Over the last years, this assay has been adapted in many ways. One question of special interest are leader cells, that often emerge at the tips of protruding fingers [22, 23]. Using microfabricated stencils to prepare monolayers of predefined shape, it has been shown that leader cells emerge from regions of high curvature [24]. Combination with the cell cycle marker Fucci in MDCK-2-cells has shown that removal of the barriers triggers release of the cells in the monolayer from cell cycle arrest [25]. Using adhesive micropatterns, it has been shown how topological defects can form when many cells have to self-organize in confined space [26]. The removable barrier approach is also increasingly combined with the micropatterning approach. A removable barrier has been used to trigger collective cell migration onto a stripe array, thus allowing the study of epithelial bridge formation as a function of ECM-geometry [27, 28]. More recently, micropatterning has been used to study the closure of non-adhesive gaps in a monolayer [29, 30]. In addition, these micropatterning techniques can also be combined with traction force microscopy, which allows us to understand the mechanical basis of wound healing [28, 29, 31, 32]. Like cell pairs, cell collectives often show rotational motion which can be quantified best on adhesive micropatterns. The rotational motion of cell collectives on circular micropatterns is observed for systems up to 200 μm in diameter but eventually disappears for larger patterns [33]. Detailed analysis revealed a dependence of the persistence time of the rotation on the number of cells and their spatial coordination on the circular island [34].

In summary, adhesive micropatterns have become a standard method to study the shape, organisation and movement of cells bridging all the scales from single cells through small groups of a few cells up to large multicellular ensembles. Although highly relevant in the three-dimensional context of physiological tissue, most of these studies have been performed for a two-dimensional setup in which the full power of microfabrication and optical microscopy can be harvested. Despite the large biological relevance and the wealth of observations made in these experimental studies, however, a unifying model starting from the single cell basis and reaching up to the collective migration of large ensembles in the presence of geometrical constraints is still missing. Here we introduce such a comprehensive approach based on a two-dimensional cellular Potts model (CPM) that is particularly suited to describe cell dynamics on adhesive micropatterns.

Motivated also by the power of quantification inherent in these experimental advances, the last decades have seen increased attention for modelling approaches to better understand cell shape, organization, mechanics and dynamics as a function of external cues [35]. An important focus of this work is the simultaneous prediction of cell shape and traction forces, using e.g. continuum mechanics approaches with contractility [9, 36–41] or discrete contractile network models [6, 42, 43]. However, these approaches are usually restricted to static cells. To address dynamic processes, the CPM is much more suitable [44–47]. CPMs generate complicated shapes with high computational efficiency and propagate them under the action of a quasi-static energy function. They were originally designed to study cell sorting [48, 49] motivated by the differential adhesion hypothesis [50]. Since then they have been widely used to address shape problems in biological systems, including e.g. packing in the *Drosophila* retina [51], gastrulation of zebrafish embryos [52] or cell packing in two- versus three-dimensional tissues [53]. They have also been adapted to describe collective cell migration, including streaming in cell monolayers [45, 54, 55], the formation of swirls [33], cell-ECM invasion [56], T-cell migration into lymph nodes [57] and the formation of endothelial networks on soft elastic substrates [58]. Closely related to CPMs are vertex models which have been used to study e.g. cell packing [59], the role of mechanical interactions in the *Drosophila* wing imaginal disk [60] and the role of contact inhibition in cell division [61]. However, vertex models can only describe cells with straight contours and are best suited to study large systems under high tension. Although they can be extended by viscoelastic elements to also describe dissipative processes, they are not suited well to describe subcellular processes such as protrusion and therefore they are usually not used to model the dynamics of single cells or small groups of cells. Another alternative to CPMs are phase field models [62–64], that recently have also been applied to cell ensembles [65, 66]. Although well suited to study the effect of micropatterning [64, 65], phase field models are computationally much more challenging than CPMs and due to their continuum character include non-local interactions.

Here we introduce a two-dimensional CPM that allows us to bridge the gap between single cell and ensemble behaviour on adhesive micropatterns with a moderate computational effort. Although originally introduced for cell ensembles, CPM have been adapted before to describe also single cell migration [67, 68] and single cell behaviour on adhesive micropatterns [69, 70]. However, they have not been used yet to explain the whole range of cellular dynamics on micropatterns, ranging from single cells through pairs or small groups of cells to large communities with collective cell migration. In the following, we will introduce our computational framework in a step-wise manner, by considering experimental situations of increasing complexity, so that each new section requires the introduction of an additional set of rules. Because all rules are part of the same comprehensive framework, new sections will always include the rules of the previous sections. Our approach for single non-dividing cells spreading and moving on adhesive micropatterns has been described elsewhere [70]. Here we first extend this approach to dividing cells and show that we obtain very good agreement with experimental results for the orientation of the cell division axis on micropatterns of arbitrary shape. We then address the issue of cell-cell interactions and introduce a novel rule for formation and rupture of cell-cell bonds. Cell migration is introduced through a reduction of surface tension at the cell front and an increase at the back (graded tension model). With these ingredients, we can accurately predict the stability of cell pairs on micropatterns. In the same framework, we then also address collective cell migration for large ensembles, including epithelial bridge formation on comb patterns and swirl formation on circular and pacman patterns. Again we obtain very good agreement with experimental data. Our model gives important new insight into the way different cellular processes contribute to overall ensemble dynamics and in the future can be used to design micropatterns for desired cell dynamics.

## Results

### Single Cell Spreading

**Fig 1:**
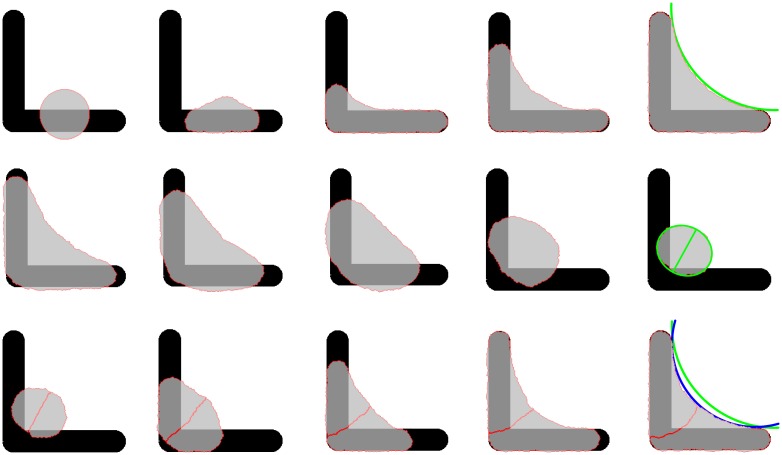
Cell spreading and division on [L] shaped micropattern. This series of snapshots first shows how a single cell spreads on the pattern (first row). It then rounds up for division (second row) and the two daughter cells distribute again over the pattern (third row). The green line in the last snapshot is the single cell arc from above and shows that the cell pair has a more invaginated contour (blue line). For a movie see S1 Movie. Model parameters as given in Table 1 (generic cell).

Our two-dimensional CPM for multicellular ensembles extends the CPM that we have developed earlier to predict single cell shape and spreading dynamics on an arbitrarily shaped micropattern (MP) [70]. The first row of Fig 1 shows the spreading process on a [L] shaped MP as it arises from our CPM (for a movie see S1 Movie). The driving force for spreading is an increase in adhesion energy as the cell (light gray) binds to more and more adhesive ligands on the adhesive substrate (dark gray). During adhesion and spreading on micropatterns, cells can dramatically change their projected area by transfering material from the third dimension into the adhesive region or back [71]. In our two-dimensional model, this corresponds to a reservoir for adhesive area that can be described by a chemical potential (with saturation at high adhesive area because adhesive area cannot grow without limits due to limitation in the influx of membrane and adhesion receptors). This approach is different from the classical CPM-formulation, that assumes that cells have a well-defined typical area (set e.g. by the density in a cell monolayer) and that deviations away from this average can be characterized by an elastic constant. Cell adhesion is only favorable as long as it does not lead to excessive mechanical energy that the cell has to spend to change its shape. The energy function and the model parameters describing this balance are given by Eq 1 and Table 1, respectively, in the Models section.

**Table 1:** Summary of the simulation parameters.

Description	Symbol	Generic cell	MCF10A	HaCaT
Surface tension	*σ*	1 nN μm^−1^	0.1 nN μm^−1^	0.03 nN μm^−1^
Simple line tension	*λ*_*s*_	10 nN	2.3 nN	2.3 nN
Arc rigidity	*k*	50 nN	40 nN	40 nN
Adhesive energy density	*W*	20 nN μm^−1^	20 nN μm^−1^	0.7 nN μm^−1^
Target cell size	*A*_0_	1300 μm^2^	1000 μm^2^	1000 μm^2^
Cadherin energy line density	*c*	-10 nN	-6.3 nN	-3 nN
Migratory strength	*μ*	2 nN μm^−1^	0.19 nN μm^−1^	0.1 nN μm^−1^
Alignment lag time	*τ*		185 MCS	40 MCS
Gradation width	*η*		0.25	0.25
Protrusion decay length	*d*_0_		43 μm	43 μm
Division rate	*r*_0_			0.0008 MCS^−1^
Hill coefficient	*m*			4
Fluctuation allowance	*T*	0.2, 0.6, 1	0.15	0.5
Lattice scale	*β*	0.1, 0.3, 0.5 $\frac{\mu \text{m}}{\text{pixel}}$	0.25 $\frac{\mu \text{m}}{\text{pixel}}$	1 $\frac{\mu \text{m}}{\text{pixel}}$
Used in Figure		1, 3, 5	4, 6, 7, 8	9, 10

The fluctuation allowance is adjusted with the lattice scale and simple tension by *T* ≈ 0.2*λ*_*s*_*β*. This choice results in similar size of membrane fluctuations for all parameters and lattice scales. When the results are independent of the exact parameter choice the generic cell is used. The parameters for MCF10A-cells are obtained from [70] and from fits to experimental data from [17] as described in the main text. The parameters for HaCaT-cells are chosen to yield a good visual agreement with experiments [28].

**Fig 2:**
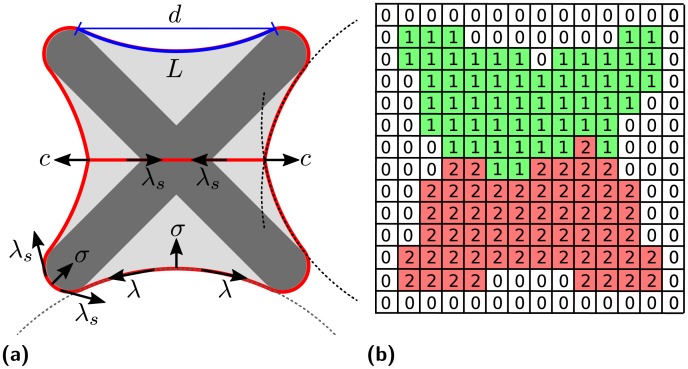
Tension and lattice representation of a cell pair. (a) Tension-based representation of a cell pair (light gray, contour in red) on a [X] shape micropattern (dark gray). All energy contributions result in forces shown as arrows. The surface tension *σ* and the simple line tension *λ*_*s*_ act everywhere along the contour (lower left). At concave parts of the patterns free spanning circular arcs form. They are reinforced by actin stress fibers resulting in an increased line tension *λ* with an elastic component determined by the arc rigidity *k* (bottom). The free spanning arcs are characterized by their radius *R*, contour length *L* and spanning distance *d* (top). Line tension along cell-cell contacts is set to the value of *λ*_*s*_ and pulls the termini of junctions inwards (left and right). At the same time, they are pulled outwards with a tension *c* accounting for the energy gained when forming new cell-cell contacts. (b) Representation of the cell pair in the two-dimensional cellular Potts model on a lattice. Empty lattice sites are indicated by the index 0. Sites belonging to the two cells are indicated by 1 and 2, respectively.

The mechanical energy stored in the cell and its final shape is determined by different kinds of tension acting throughout the cell as illustrated in Fig 2a [6, 35, 70]. The surface tension *σ* mainly results from actomyosin contractility in the cell cortex and wants to reduce cell area. At the cell periphery, cortex and membrane fold back onto themselves, resulting in a simple line tension *λ*_*s*_ that wants to the draw the contour straight. Mathematically, the interplay between these two tensions leads to a Laplace-type law and circular contours [6]. Motivated by experimental results for cell shape on dot-like micropatterns [6, 7], here we implement the tension-elasticity model that attributes an additional elastic energy to freely spanning arcs because they are reinforced by actin cables with a strong elastic response. The elastic arc rigidity *k* (compare Eq 1) leads to a line tension (defined as the derivative of the energy function with respect to the local cell contour length) that is increased from the simple line tension *λ*_*s*_ to a larger line tension *λ* along the arcs. The line tension *λ* is different for each arc and the resulting forces are shown schematically for the bottom arc in Fig 2a. Because it is the same for each point within a given arc, the Laplace law still holds and all arcs are circular, as also seen in the last image of the top row in Fig 1.

Cell-cell junctions are contractile and pull the outer contour further inwards, compare the last snapshot in Fig 1 and the left and right arcs in Fig 2a. For simplicity and as explained in more detail below, here we assume that the resulting line tension at cell-cell boundaries equals the simple line tension *λ*_*s*_ of cell-matrix adhesion. Finally we account for an energy gain that comes with the formation of new adherens junctions. This corresponds to an additional line tension *c* pulling the endpoints outwards. Due to the force balance at these positions, the outer cell contour has a kink as revealed by the dashed lines fitted to the right circular arcs in Fig 2a.

The different parameter values of our model can be fitted from experimental results for cell shape and forces for specific cell lines of interest [70], e.g. for breast epithelial MCF10A-cells on fibronectin patterns [8, 17] or keratinocyte-derived HaCaT-cells that form epithelial bridges during wound healing [28]. These results are given in Table 1. In Fig 2b we show schematically how a cell pair is represented by two connected domains in the two-dimensional CPM. In our simulations, each cell is typically represented by 10^4^ lattice sites. Using a refined marching square algorithm, cell area and perimeter can be calculated with very high accuracy (for a circular cell of this size, area and perimeter typically deviate by 0.1% and 1.5%, respectively). In contrast to other implementations of the CPM [53], we do not have to use a mapping between computed and actual perimeter values and therefore the line tensions can be compared directly to experiments.

### Cell Division

The orientation of the cell division axis is strongly influenced by the cell shape prior to division [13–15]. Previous models were able to reproduce division plane orientations correctly for adherent HeLa-cells [14] by relating the pattern geometry to a torque applied to the dividing cell. However, this model required an additional fit for each adhesion pattern and therefore lacks predictive capability for unknown patterns. Here, we introduce an alternative approach allowing predictions on arbitrary geometries without any parameter fit. Our approach mimics cell rounding and detachment during mitosis [72] and is motivated in detail in the Models section. Briefly, we let the cell contract by an increased line tension while all contacts between the cell and the matrix are disassembled. Inspired by the long axis rule [73, 74], we fit an ellipse to the cell during the contraction when the cell area is contracted to *r* = 0.36 of its original size. The major axis of this ellipse is chosen as the division axis. With this approach the complex cell shape is mapped onto a single direction. Noise inherent to our model through the Monte Carlo steps of the CPM result in statistical variations of this direction as we perform typically 20.000 simulations for each pattern of interest. In experiments, the cell division orientation is typically determined by imaging the mitotic spindle and therefore we use the term spindle orientation synonymously for cell division axis.

**Fig 3:**
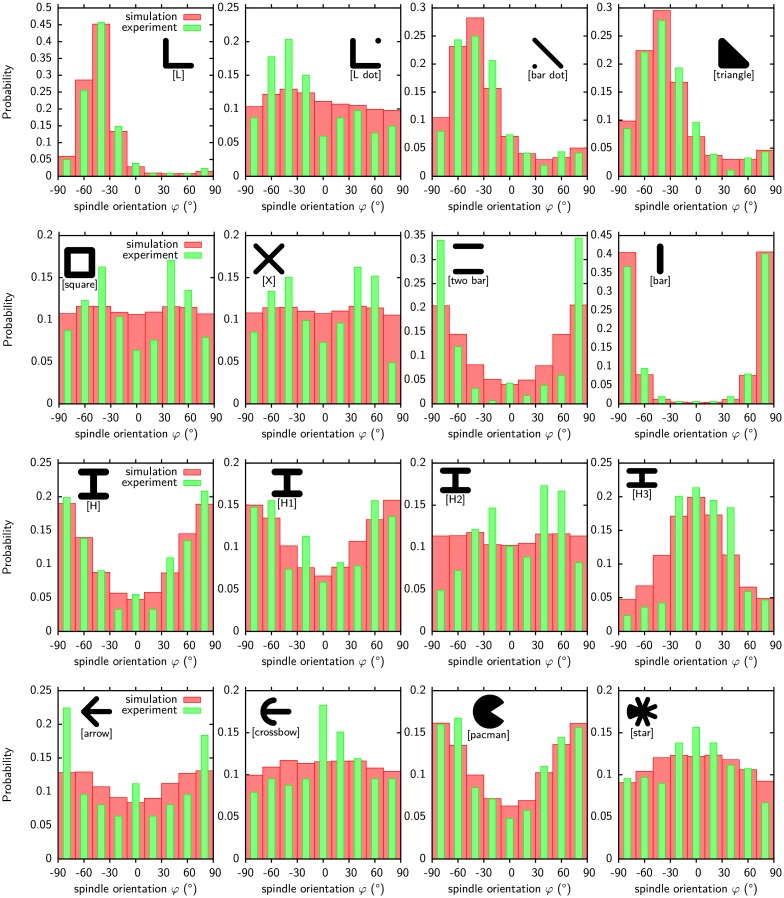
Orientation of cell division. Comparison of predicted (red, from ellipse fitting) and experimental (green, from mitotic spindle) orientations for various micropatterns. The experimental data is for HeLa-cell [14]. The histograms show the statistical distributions of the orientation of the cell division axis. In our two-dimensional model, this orientation is given by the polar angle (zero for the x-axis). For each pattern 20.000 cell divisions were simulated, resulting in the observed variability. Model parameters as given in Table 1 (generic cell).

The middle row of Fig 1 shows snapshots of a dividing cell with an ellipse fitted to the contour once the prescribed area ratio *r* = 0.36 has been reached. The shape transformation predicted by our model from a spread cell to a round one is very similar to the transformation observed for HeLa-cells [13]. Fig 3 shows that the resulting distribution of cell division axes (or, equivalently, spindle orientation, measured by the polar angle to the x-axis) agrees very well with the experimental results for HeLa-cells [14]. E.g. for a cell dividing on [L] it is most likely for the division axis to have an orientation of −45° and the two daughter cells are located on the two arms of the [L] shaped pattern as shown in Fig 1. Another simple example is the [bar] pattern, where the division axis is most likely at ±90°, corresponding to the two daughters being positioned at the top and bottom ends of the bar.

Several key aspects are predicted correctly by our model. For [L dot] and [H], different distributions are predicted although both patterns have the same convex hull and similar cell shapes. This indicates that the location of the invaginated arcs influences the division plane. The model also predicts the rotation of the division axis by 90° correctly when the pattern is changed from [H] to [H3]. This rotation is shown for more aspect ratios of the [H] pattern in S1 Fig. The model also accounts for the strong broadening of the distribution when [L] is modified to [L dot]. The symmetry of the distribution is also predicted correctly. The patterns in the two central rows have a two fold mirror symmetry (except [square] and [X]) and one of the symmetry axis is selected as the main division axis. Patterns in the top and bottom row only have a one-fold mirror symmetry. However, the symmetry axis of the pattern does not set the main division axis as it can be seen for [pacman] and [star]. Both patterns have the same orientation of the symmetry axis but the most likely orientation is turned by 90°.

We also note some small deviations between our predictions and the experiments. [H] and [two bar] differ only by the central bar and the distributions for our cells are very similar while experiments show a more strongly peaked distribution on [two bar]. The two patterns differ in size, [H] has a width of 36μm and [two bar] one of 30μm. It is not clear if the difference of the distributions comes from the size difference, i.e. that the [H] pattern is too large for the cells, or the absence of the central bar. For the simulations it was assumed that our cells can cover the complete [H] pattern. For the [arrow] (width 48μm) and [crossbow] (width 38μm) the size difference is even larger and the observed trend in the simulations to rotate the division axis by 90° is only due to this size difference. The [arrow] patterns requires cells with an area of 2000μm^2^ to be fully covered. Our cells have an area of 1300μm^2^ not fully covering the pattern and they localize to the tip of the arrow which elongates them perpendicular to the symmetry axis giving rise to the observed orientations.

The distributions predicted by our model as shown in Fig 3 are almost independent of the parameters defining the cell shape, namely surface tension *σ*, simple line tension *λ*_*s*_ and arc rigidity *k*. The agreement between simulated and experimental distributions when the shape defining parameters are changed is quantified in subfigures (a) and (b) of S2 Fig. This figure shows that the optimal area ratio *r* = 0.36 to fit the ellipse does not depend on the parameters. Only when the surface tension is large compared to the line tension do deviations occur. Cells are then no longer able to spread across the gaps on [L dot], [bar dot] and [two bar] and the different shapes before division result in poor agreement with experimental predictions for those patterns. Subfigure (c) of S2 Fig shows how the division plane is linked to shape fluctuations. If one allows for larger fluctuations, then one has to fit the ellipse earlier, because otherwise all information about the shape prior to division is lost. In general, fitting the ellipse early during contraction results in strongly peaked distributions and fitting it late in almost uniform ones. Subfigure (d) of S2 Fig shows that residual adhesion remaining during contraction worsens our predictions.

### Cell-Cell Contacts

After division two cells adhering to each other reside on one MP as shown in the bottom row of Fig 1. As described in detail in the Models section our approach to cell-cell adhesion differs from earlier versions of the CPM. Cell-cell adhesion is mediated by cadherins which are transmembrane proteins forming adherens junctions between cells [75]. Many models treat cell adhesion by a reduction of the line tension at cell-cell interfaces opposed to cell-medium interfaces [48, 49, 51, 52, 76]. The formation and breakage of adherens junctions releases and requires energy, respectively. Therefore, in an energy-based description cells can lower their total energy by increasing the length of cell-cell contacts. This is equivalent to assuming a reduced line tension at cell-cell boundaries.

Earlier it has been argued that cortical contractility is equally important as is adhesion energy to determine cell-cell adhesion [52]. Cortical tension is reduced close to adherens junctions and this can have different effects than a change in adhesion energy only. The question then arises how to separate adhesion- and tension-based mechanisms in a energy-based description. Here we suggest that line tension should be kept high in order to avoid floppy configurations that are suppressed by contractility. In particular, negative values should not be allowed for the effective line tension *λ*. Further we suggest that the driving force for contact formation is mainly localized to the endpoints, as observed experimentally [17]. Thus energy is only gained when previously unconnected parts of two cells come into contact, but not if the existing contact line elongates by a pure deformation which does not form new contacts (compare S4 Fig). Our approach is equivalent to applying an outward directed force to the endpoints of a junction as shown in Fig 2a.

**Fig 4:**
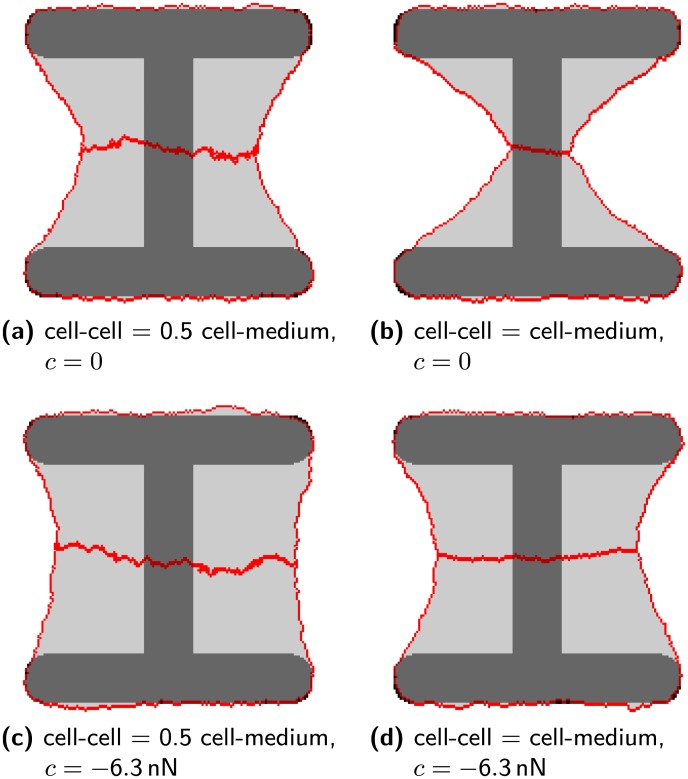
Difference between line tension and contact driven cell-cell adhesion. (a) The line tension at the cell-cell contact is set to half the tension of the cell-interface resulting in strong fluctuations of the cell-cell interface. The cadherin energy density *c* is zero. (b) Cell-cell line tension and cell-medium line tension are the same, *c* = 0. The cell-cell interface has the same kind of fluctuations as the cell-medium interface, but the cell-cell adhesion is too weak to extend the cells above the non-adhesive areas. (c) as (a) but with *c* = -6.3nN. The low cell-cell tension results in strong fluctuations and the cadherin based adhesion extends the contour further above nonadhesive areas. (d) as (b) but with *c* = -6.3nN. Cell-cell and cell-medium tension are the same resulting in a cell-cell interface with little fluctuations. The cells extend above the nonadhesive areas due to the cadherin based adhesion. The other parameters are that of MCF10A-cells, compare Table 1.

Fig 4 shows two cells with different values for contractility and adhesion energy. The energy line density associated with the cadherin bonds is denoted by *c*. This quantity equals the number of cadherin molecules per length times the energy associated with each bound molecule. It is assumed to be of the order of several thermal energies per cadherin. Without an explicit treatment of cadherins (*c* = 0, top row of Fig 4), adhesion is only controlled by differences in the tension between cell-cell and cell-medium contacts. MCF10A-cell pairs on [H] pattern spread almost fully across the non-adhesive regions [17]. To achieve such spreading the tension at the cell-cell interface must be reduced to at least half the level of the cell-medium tension as shown in Fig 4a. This results in a strongly fluctuating interface between cells. By measuring the resistance to deformation it should be possible to estimate the line tension at the interface experimentally. Naively one would expect the tensions to be higher there because the cortices of both cells contribute to it. Setting the interface tension to the same value as the cell-medium tension results in no spreading across the non-adhesive areas (Fig 4b). The cell junction is too contractile in this case. With explicit treatment of cadherins at the endpoints (*c* < 0, bottom row of Fig 4) cells spread out more easily. A reduced tension still results in strong fluctuations at the interface (Fig 4c). Setting cell-cell and cell-medium tension equal now allows spreading (Fig 4d). The junction remains contractile and flat in this case. The contractility of junctions can also be seen by comparing the contour of the single cell and cell pair in Fig 1. The last frame shows two circles, the blue one is fitted to the contour of the cell pair and the green one is fitted to the contour of the single cell. The radius of the blue circle is smaller indicating a higher contractility of the cell.

### Single Cell Migration

**Fig 5:**
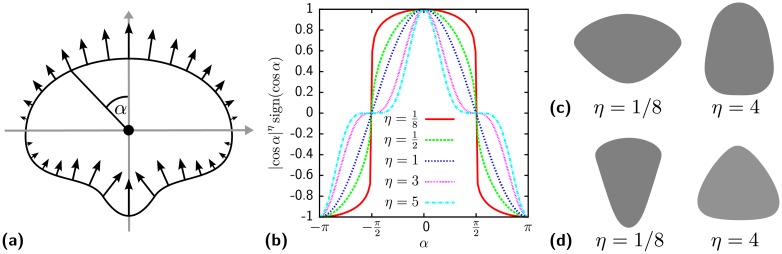
Tension generated by the migratory machinery acting on the cell contour. (a) Cell polarized along the y-axis with center of mass in the origin of the coordinate system. The arrows are oriented perpendicular to the cell contour. They either represent displacements as in the graded radial extension model [77] or forces acting on the contour. The angle *α* is defined between the polarity direction pointing upwards and a point on the contour. (b) Strength of the persistence tension along the contour. The shape of the gradation is varied by the parameter *η*. For *η* < 1 the distribution becomes broad and for *η* > 1 it becomes peaked. (c) Cell shapes predicted by the CPM for different gradation widths. The polarity direction of the cells was kept fixed pointing up and the cells shapes where averaged over 10^5^ Monte Carlo sweeps. A broad gradation with *η* = 1/8 results in a keratocyte shaped cell and a narrow gradation with *η* = 4 in a neutrophil shaped one. (d) To model actin polymerizing in one direction with a uniform front instead of a radial dependance, we replace the polar angle *α* by the angle between the normal of the contour and the polarization direction. Then the cell resembles a fibroblast for *η* = 1/8. The shape obtained for *η* = 4 does not resemble any model cell type. Model parameters as given in Table 1 (generic cell).

Migration of crawling animal cells is driven by actin polymerization pushing against the membrane at the front and myosin contractility pulling at the back. We describe both processes through a graded tension model described in detail in the Models section. Fig 5a demonstrates how the tension generated by the actomyosin activity is distributed spatially inside the cell. At the cell front actin polymerizes in a direction normal to the contour pushing it forward. The resulting forces are indicated by arrows and decrease gradually towards the sides of the cell because of decreasing polymerization activity. At the rear of the cell myosin contraction results in inward directed forces which are assumed to have the same magnitude as the actin generated forces. The combination of actin and myosin forces result in a reduction of the surface tension *σ* by *σ*_*m*_ = − *μ* cos(*α*). The parameter *μ* controls the strength of the migration machinery and the cosine the gradation towards the sides of the cell. The angle *α* is measured with respect to the polarization direction and defines the location of a point on the cell contour. As shown in Fig 5b the width of the gradation can be controlled by taking powers of the cosine function. For powers *η* < 1 the gradation is wide and protrusion forces at the center of the leading edge (*α* = 0) stay relatively constant and decay rapidly at the sides of the cell (*α* = ±*π*/2). For *η* > 1 the gradation is sharply peaked at the center of the leading edge. The width of the gradation has a strong impact on the cell shape of migrating cells. A broad gradation (*η* < 1) results in a keratocyte shaped cells while a narrow one (*η* > 1) results in cells resembling neutrophils (Fig 5c).

The shape of the migrating cells is defined by the balance of forces arising from the line and surface tension and the migratory tension *σ*_*m*_. At curved region of the contour the line tension results in a force perpendicular to the contour [70]. For concave shaped regions this force is directed inward. Its magnitude increases with increasing curvature. To maintain a stationary shape during migration, the forces generated through curvature, surface tension and migratory machinery must add up in a way which allows to translocate the cell without changing its shape. For *η* = 1 this shape is a circle as in the graded radial tension model [77]. For a circular shape, curvature induced forces are irrelevant because they always point to the center with constant magnitude along the contour. The resulting anisotropic tension responsible for translocation is the bare migratory tension *σ*_*m*_ = cos(*α*). It is proportional to the force acting on the contour which is, in turn, proportional to the velocity of the contour. Hence, the velocity is *v*(*α*) ∝ cos(*α*) which is the velocity required to propagate a circular contour without deforming it [78]. For gradation deviating from the simple cosine the shapes of Fig 5c arise. The broad gradation and strong protrusive forces for *η* < 1 are ultimately stalled by the high curvature at the sides of the lamellipodium. Viewed from a different perspective, strong changes of the gradation allow for changes of the curvature.

Our gradation model assumes that actin polymerizes normal to the membrane with its activity decreasing towards the sides of the cell. If actin is assumed to polymerize uniformly in the polarization direction it pushes against the membrane with an angle reducing its force. The angle is given by the normal of the contour with respect to the polarization direction. The resulting shape of cells resemble fibroblasts as shown in Fig 5d for *η* < 1. We speculate that such an additional polarization effect might arise from the presence of stress fibers. For *η* > 1 this acting protrusion mechanism generates triangular cells which seem to have no biological counterpart.

Single cells can maintain their polarity direction and move along it over a distance of several hundred of cell diameters [79]. Directionallity in cells migrating collectively in sheets can also stay correlated over several hours [18]. On longer time scales their movement becomes random. To control the polarity direction and persistence time we use the well established velocity alignment model [45, 65, 80–82] where cells try to align their polarity direction with their current movement direction. Polarity and movement direction do not need to be the same. Misalignment between them can occur through fluctuations of the center of mass movement, boundaries imposed by the MP or other cells which do not allow further movement along the polarity direction. The central parameter of the velocity alignment model is the lag time *τ*. It controls how fast the polarity direction aligns with the current movement direction. As discussed in the Models sections this model can be interpreted as cells keeping a memory of their past movement. The lag parameter *τ* sets the timescale for how long past steps are remembered. A typical cell trajectory obtained with this model is shown in subfigure (a) of S3 Fig. The implicit noise originating from the fluctuation allowance leads to a coupling between cell speed and persistence time as shown in subfigure (b) of S3 Fig. The persistence time first increases exponentially with speed. The increase saturates for higher speeds as observed for many different cell types [12].

### Rotation of Cell Pairs

We next turn to the migration of small groups of cells on MP. Special attention is needed when cells contact each other or encounter the border of an adhesive area of the substrate. Cells retract their protrusions and change directions when they encounter another cell [83]. This contact inhibition of locomotion helps cells to coordinate their migration collectively. We implement it by preventing any protrusive activity when a cell would invade another cell.

Protrusions generated by polymerizing actin need the actin network to be anchored to the substrate which occurs a few microns behind the leading edge [78]. Above non-adhesive areas the anchoring support is missing and it is assumed that the polymerizing actin mesh can only reach a certain extension before polymerization just pushes the mesh backwards without extending the boundary. This is put into effect by reducing the migratory strength *μ* with distance from the adhesive substrate as described by Eq 4.

**Fig 6:**
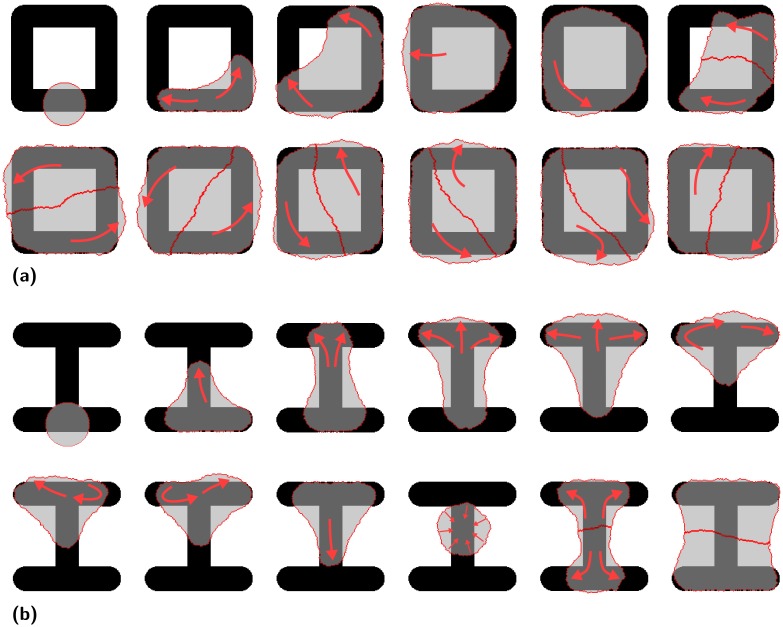
Cell pairs migrating on [Square] and [H] micropattern. (a) An initially round cell is spreading on a [square] pattern. The migration direction is indicated by arrows. The single cell oscillates around the corners until it divides. The resulting cell pair rotates persistently switching directions from time to time. For a movie see S2 Movie. (b) Same as (a) but on [H] pattern with more frames in the single cell phase. A single cell on an [H] pattern is unstable and oscillates from top to bottom. When the cell divides the resulting cell pair is stable with the cell-cell junction positioned above the nonadhesive part of the substrate. For a movie see S3 Movie. Model parameters as given in Table 1 (MCF10A-cells).

With this model we can now predict behavior of cell pairs on MP. Snapshots of our simulation are shown in Fig 6 (also see S2 and S3 Movies) with the parameter values for MCF10A-cells. Indeed our results agree very well with the experimental observations for this cell line [17]. In Fig 6a a single cell initially spreads on a [square] pattern. The pattern is too large to be fully covered by the cell and it finds no stable position. It oscillate in a circular fashion between the corners of the pattern until it divides. The two daughter cells adhere to each other and start to rotate. At the leading edge a lamellipodium extending beyond the pattern is visible. The rotation direction is reversed from time to time as experimentally observed for MCF10A-cells [17]. In marked contrast, cell pairs on [H] patterns do not rotate. They remain in a stable configuration with the cell-cell junction positioned above the nonadhesive region. However, single cells on [H] patterns are highly mobile as shown in Fig 6b. They mainly oscillate from top to bottom (compare S3 Movie) but also from left to right. In general, the stability of a cell pair strongly depends on the size of concave regions. Cells on [C], [H] and [hourglass] pattern are stationary while they move on [square] and [C2] [17]. This suggests that rotation is larger if the convex hull of a pattern is adhesive [17]. In the framework of our model this can be understood by the requirement of an adhesive area near the leading edge to support actin polymerization and by the contractility of the cell-cell junctions. A minimal energy state for a cell pair is usually found when the length of the cell-cell junction is minimized. For the pair on the [H] pattern shown in Fig 6b this results in the junction being above the non-adhesive part of the substrate as every other configuration would make it longer. A minimal junction length minimizes the contribution of the line tension to the cell energy. On patterns with a continuously adhesive hull such as [square], junctions cannot be shortened by placing them above non-adhesive regions.

For cell pairs to rotate junctions must be stretched and reach at least the diagonal extension of the patterns which is energetically unfavorable. The required energy comes from the migratory machinery. Large changes in junction length and therefore large energy differences make it unlikely that the cells maintain their polarity long enough to cross the energy barrier imposed by a diagonally oriented cell-cell junction. Persistent rotation can be promoted by increasing the migratory strength *μ* or the lag time *τ* which makes it easier to overcome the barrier. Alternatively the simple line tension *λ*_*s*_ can be decreased.

**Fig 7:**
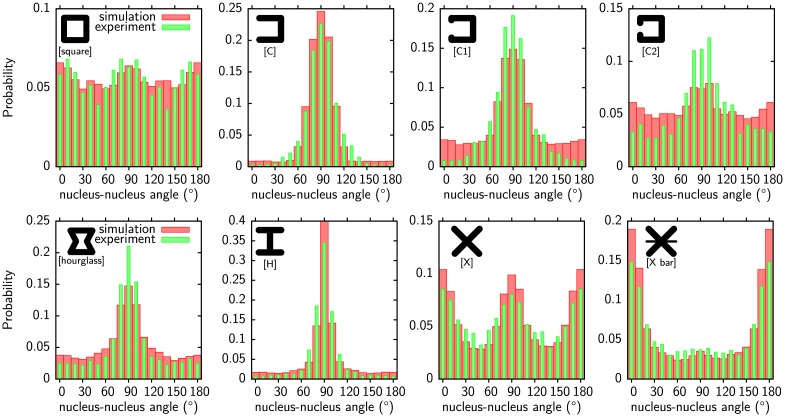
Experimental and theoretical histograms of the nuclei-nuclei axis orientation of cell pairs. Comparison of predicted (red, from direction between centers of mass) and experimental (green, from Hoechst staining) [17] nucleus-nucleus axis orientations. The experimental data [17] was converted from polar histograms into Cartesian coordinates. The same parameter set as for Fig 6 was used.

Fig 7 shows in green distributions of the nucleus-nucleus axis orientations obtained for MCF10A-cells on various patterns [17]. On all patterns orientations with the shortest cell-cell junctions are most likely (the cell-cell junction is usually perpendicular to the nucleus-nucleus axis) but the degree of bias towards the shortest junctions varies. Among the shown patterns [H] offers the possibility of the shortest junction and has indeed the most sharply peaked nucleus-nucleus axis orientation. It is followed by [C] where the minimal junction length is slightly longer opposed to [H] because one more side is adhesive and the contour is not invaginated there. The longer minimal junction length results in a broader peak. This trend continues for [square] where now all sides are adhesive and the minimal junction length is set by the width of the pattern.

The most relevant model parameters for persistent cell rotation are the shape-defining parameters: surface tension *σ*, simple line tension *λ*_*s*_, arc rigidity *k* and the cadherin energy line density *c*. These parameters describe how strongly cells are invaginated at concave parts of the pattern. The migratory machinery strength *μ* and lag time *τ* act as a counterpart. They set how much force is available for the cells to extend their cell-cell junction and for how long this force is applied.

From traction force measurements the values of the surface tension *σ* = 0.83nN μm^−1^, the simple line tension *λ*_*s*_ = 2.3nN and the arc rigidity *k* = 40nN are known for MCF10A-cells [70]. However, cells are too strongly invaginated to allow persistent rotational motion with these parameters. Agreement with experiments can only be obtained if the surface tension is lowered. We keep the values of the simple line tension and the arc rigidity fixed and fit the other model parameters to the experimental distributions for MCF10A-cells. The fit is performed by minimizing the least square deviation between experimental and predicted histograms with Powell’s method. Excellent agreement results as shown in Fig 7. Our model has no explicit description of a cell nucleus and we take the center of mass instead. The choice of the gradation width *η* has no influence on the agreement between simulation and experiments. It only influences the other fit parameters and we take *η* = 1/4 which results in cells with a broad lamellipodium. The optimal surface tension is given by *σ* = 0.1 nN μm^−1^ which is significantly lower as previously reported [70]. The best fitting values for the migratory machinery are *μ* = 0.19 nN μm^−1^ and *τ* = 185 *MCS* (Monte Carlo Sweeps) resulting in a persistence length of 40μm defined by angular correlations. The scale on which the protrusive activity decays is found to be *d*_0_ = 43 μm. Although this value is large, lamellipodia extend only about 5 μm beyond the adhesive substrate. The protrusive activity is mainly stalled by the line tension. For narrower gradations (*η* > 1) this decay length is more important and becomes shorter. The migratory tension is larger in this case. The optimal cadherin adhesion energy line density was found to be *c* = -6.3 nN which results in an area density of 12.6 nN μm^−1^ if the cell junction is assumed to have a height of 0.5 nN.

**Fig 8:**
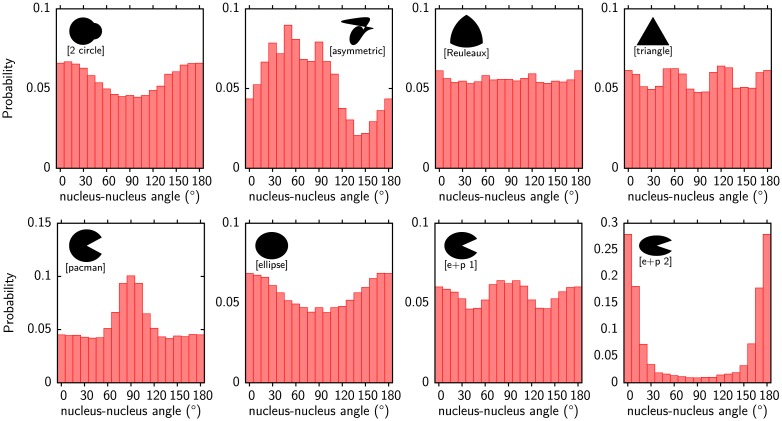
Predicted histograms of the nuclei-nuclei axis orientation of cell pairs. Orientations are identified with the directions of the centers of mass. Cells on [2 circle] do not obey the pattern symmetry for the most probable orientation. Instead the aspect ratio determines it. Distributions on arbitrary geometries can be predicted, as shown here for the [asymmetric] pattern. A [Reuleaux] triangle is a curve of constant width. Cells on it rotate similar to a circle indicated by a uniform distributions. Cells on a [triangle] deviate from the uniform distribution. Cell on [pacman] shaped patterns position their junction most likely above the non-adhesive wedge. On an [ellipse] the junction is most likely along the shorter semiaxis (aspect ratio 0.83). Combining [pacman] and [ellipse] into [e+p 1] results in a superposition of both distributions. Increasing the aspect ratio even further (ratio 0.625) in [e+p 2] makes the wedge unimportant. Model parameters as given in Table 1 (MCF10A-cells).

We can now investigate migration of cell pairs on arbitrary MP which have not been studied in experiments before. In Fig 8 we show some representative examples. This includes cells on a [2 circle] pattern which do not obey the symmetry axis of the pattern or completely asymmetric patterns as the [asymmetric] layout which results in a non-smooth asymmetric nucleus-nucleus axis orientation. We can also test layouts which further support our idea that the observed distributions can be explained by the contractility of the cell-cell junctions alone. One candidate is a Reuleaux triangle which is a triangle with circular edges. A Reuleaux triangle is a curve of constant width meaning that every line crossing the triangle through its center has the same length. For the rotating cell pair this means that the cell-cell junction always has the same length. The pair should rotate as cells on a circular pattern although the cells change their shape during rotation on the Reuleux triangle. The resulting nucleus-nucleus axis distribution shown in Fig 8 are almost uniform confirming the importance of the junction contractility. Deviations most likely arise because the junction is not always straight. The distribution on a regular [triangle] deviates significantly from a uniform distribution as also shown in Fig 8. The shapes of both cells are very similar, one cell is usually triangular having contact with one corner while the other is rectangular having contact with two corners (compare S4 and S5 Movies). Cells on the regular triangle also switch their rotation direction more frequently. The behavior of cells on the triangles strongly supports the importance of the junction contractility.

The relative importance of shortening a junction by positioning it above a non-adhesive area or by selecting the shortest connection above adhesive parts can be investigated with [pacman] and [ellipse] patterns. On a [pacman] pattern the most likely orientation is with a horizontal oriented junction while the junction on a [ellipse] is oriented vertically along the shortest semiaxis (compare bottom row Fig 8). A combination of both patterns into an ellipse with a nonadhesive wedge ([e+p 1]) results in a superposition of the [pacman] and [ellipse] distributions. Increasing the aspect ratio further ([e+p 2]) shifts the distribution in favor of the ellipse.

The reduction of myosin activity by blebbistatin revealed a stronger reduction in surface tension than in line tension for rat embryonic fibroblasts [7]. Thus, cells on concave patterns increase their size when myosin activity is reduced. A similar reduction in surface and line tension for MCF10A-cells described by our CPM results in broader distributions as shown in S5 Fig. The increased cell area make cell junctions above nonadhesive areas longer. They need to be extended less for rotation which results in the broader distributions. For [square] and [C2] the distributions stay the same because the junction length is not changed on these patterns by inhibition. For [hourglass] patterns the broader distribution has been observed before [17].

### Collective Cell Migration

**Fig 9:**
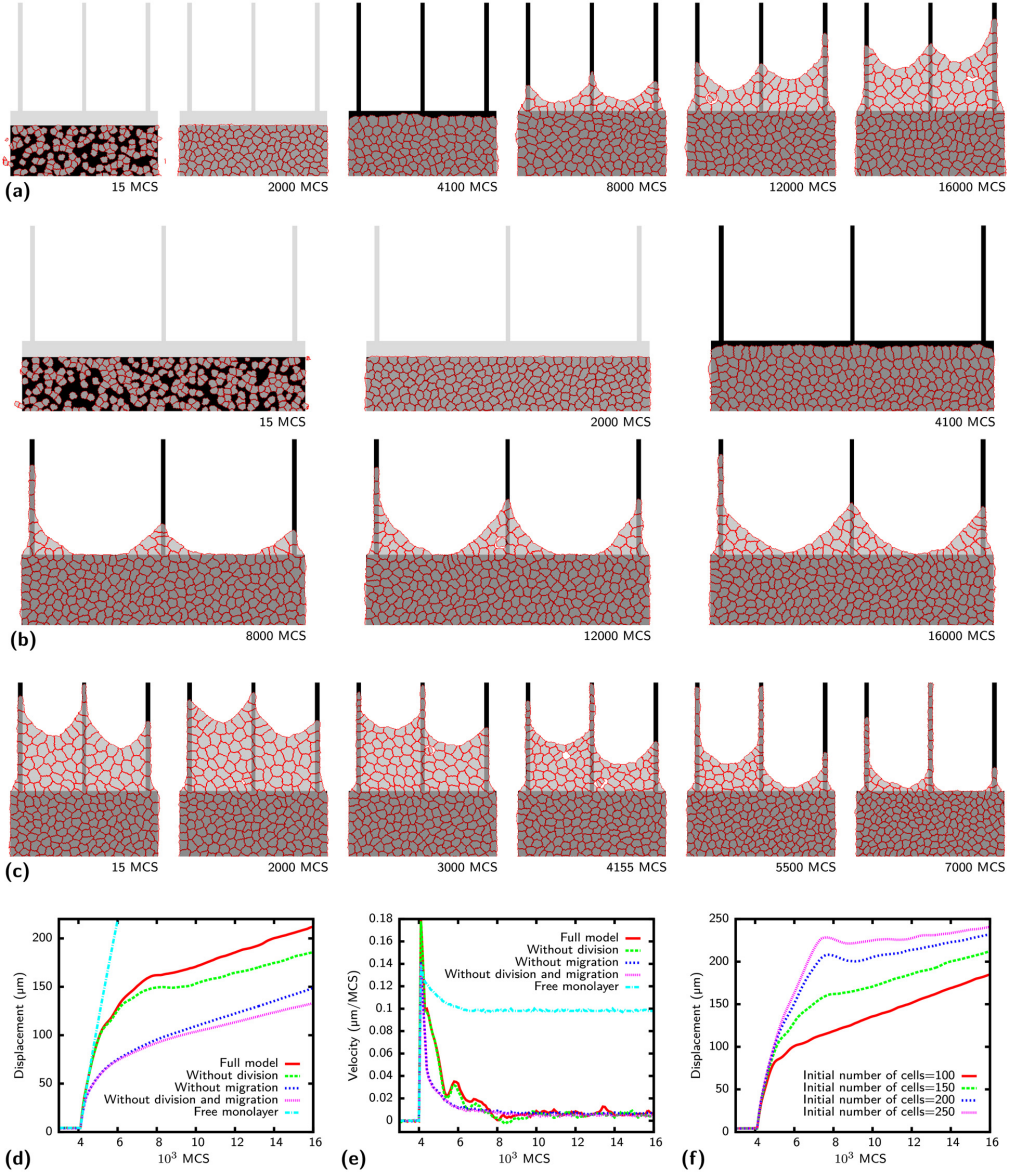
Collective cell migration. Cells are initially confined to the rectangular reservoir and cannot migrate to the gray parts of the pattern until the barrier is removed. When the confinement is released at 4.000 Monte Carlo sweeps (MCS) cells migrate fast across the newly accessible area. Migration along the 10 μm wide stripes is slowed down by the contractile bridge formed between the stripes. Dividing cells can result in holes. Stripe distance 120 μm, initial number of cells: 150, final number 225. (b) When the stripe distance is increased to 240 μm no bridges are formed. Initial number of cells: 280, final number: 370. (c) Knockdown of cadherins by a reduction of the cadherin line energy density from *c* = -3 nN to *c* = -1.5 nN after 2000 MCS results in a collapse of the bridge. (d) Temporal evolution of the displacement along stripes of a [comb] pattern and a free monolayer. Simulations without cell division or cell migration are compared. The average over 500 simulations performed on the pattern of part (a) is shown. (e) Velocity of monolayer and of cells on [comb] pattern along stripes. The velocity of actively migrating cells fluctuates after confinement removal. (f) Effect of initial cell number on displacement. Model parameters as given in Table 1 (HaCaT-cells).

MP are increasingly used to investigate collective cell migration, often in combination with removable barriers. We first investigate the formation of epithelial bridges on a comb pattern when cells are allowed to migrate from a reservoir onto a stripe array such that they have to bridge over the intervening spaces in order to advance. Fig 9a shows cells initially confined to a rectangular reservoir. The cells spread, migrate and divide forming a monolayer. Contact inhibition decreases the proliferation rate in denser tissues [61] as explained in the Models section. With the high initial density shown in Fig 9a divisions are not frequent. The cells are highly mobile and form swirl-like structures (see S6 Movie) until the constraint confining them to the rectangular area is removed after 4.000 MCS. Without confinement cells migrate fast to the newly available reservoir region until they reach the adhesive stripes. The movement of the cell front slows down and an epithelial bridge is formed between the stripes as observed experimentally for HaCaT-cells [28]. Extension of the bridge is driven by cell division inside the bridge, cells migrating into the bridge region and pulling of the leading cells on the bridge. Dividing cells loose contact to their neighbors which can form holes (e.g. at 12.000 MCS). Migration into the bridge region is slower compared to migration on a homogeneously adhesive substrate because cells tend to keep contact with the substrate. Increasing the distance between the stripes from 120 μm in Fig 9a to 240 μm in Fig 9b prevents the formation of bridges as observed experimentally for HaCaT-cells [28] (see S7 Movie). The migration along the stripes is limited and the leading cells are closer to the reservoir. Inhibition of adherens junction by decreasing the cadherin line energy density from *c* = -3 nN to *c* = -1.5 nN results in retraction of the bridge as shown in Fig 9c (see S8 Movie). With less cadherin activity balancing the contractile junctions the leading cells are too weak to pull the cell sheet outward. In addition, cells are less cohesive and cell division can result in larger holes as observed for HaCaT-cells [28]. Bridge extension can also be stopped when the stripes are not parallel but tilted as shown in S6 Fig (compare S9 Movie). When the cell sheet moves along the stripes the distance between stripes increases which slows down progress in the bridges.

Our cellular Potts model allows us to separate the contributions of cell division and migration to bridge formation. Fig 9d shows the displacement of the cells along the stripes as a function of MCS averaged over 500 simulations. Without cell division (*r*_0_ = 0, green curve) cells advance very similar as the full system at first until the bridge formation starts at ≈ 6.000 MCS. Without cell division in the bridges further progress is slightly slowed compared to the full model. Setting the migratory strength *μ* to zero has a much stronger effect. Without the active movement it is more difficult to pull cells into the non-adhesive regions slowing the overall progress. Additional removal of cell division further decreases the advance, but the effect is rather small. Compared to a freely migrating monolayer on a continuously adhesive substrate progress along the stripes is rather slow as observed experimentally [28]. Fig 9e quantifies the previously mentioned slowdown when cells start to move onto the stripes. The velocity oscillates for actively migrating cells as observed experimentally [28]. Responsible for this oscillation is the feedback mechanism in the velocity alignment model. Cells move along the stripes until further advancement is stalled when cells have to be pulled into the nonadhesive region. Without continuous progress in one direction the polarity vector becomes random and the migration direction can even be reversed. However, backward migrating cells turn around fast when they are stopped by the bulk and advance on the stripes again. Repetition results in the oscillations of the velocity. The amplitude decays because the oscillations get out of phase and 500 simulations are averaged. The cell density in the confined region also has an impact on the migration along the stripes as shown in Fig 9f. A higher initial number results in smaller cells which are biased more strongly towards free adhesive areas on the stripes making migration faster. The oscillatory behavior is also more pronounced for a larger number of initial cells and migration can even be reversed for a short time.

**Fig 10:**
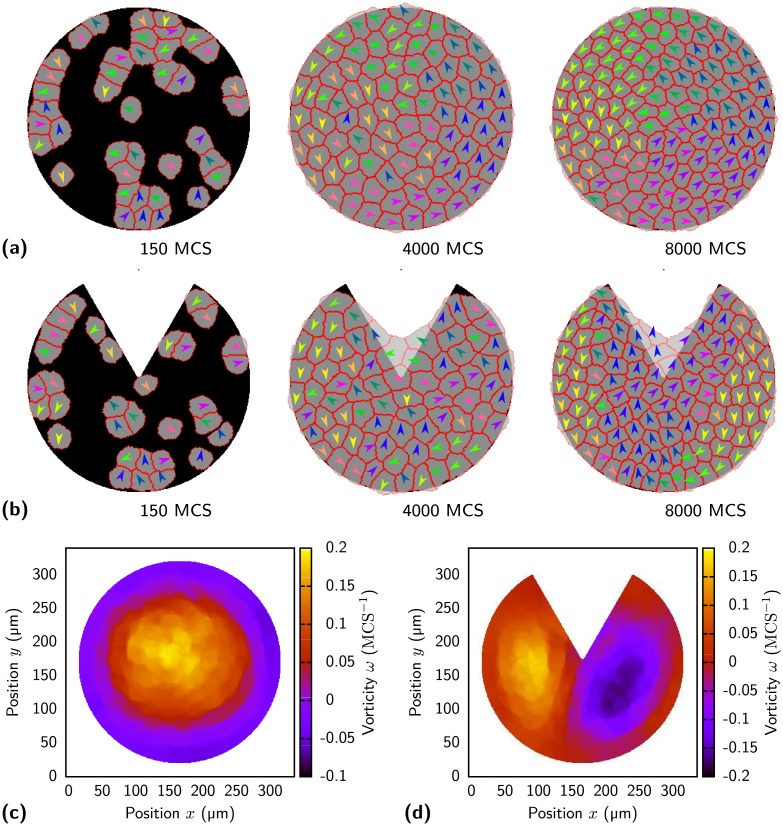
Swirl formation on circular and pacman patterns. (a) Cells on a [circular] pattern (width 300 μm) with their polarity direction indicated by colored arrows. Arrows pointing in a similar direction have a similar color. Cells move randomly at first but their movement becomes swirl like for higher cell densities. (b) On a [pacman] pattern two swirls are formed. (c) Vorticity calculated for each lattice site of the [circle] pattern after 8.000 MCS. The vorticity is averaged over 750 MCS. A global counterclockwise rotating swirl of cells is indicated by the large area of positive vorticity at the center of the pattern. Cells at the border have a lower angular velocity resulting in a negative vorticity. (d) Vorticity for [pacman] pattern. Two swirls rotating in opposite directions are visible. Model parameters as given in Table 1 (HaCaT-cells).

Circular MP have been demonstrated to promote the formation of a single swirl [33]. We demonstrate here that modifications to the circular geometry can result in two swirls. With a low initial cell density on a [circular] pattern cells divide frequently and move in an uncoordinated fashion as shown in Fig 10d (see S10 Movie). When the cell density increases division slows down and the cells start to move in one large swirl as observed for MDCK-cells [33]. The effect of the population size for the formation of a swirl has been pointed out before [45]. The single swirl can be split into two swirls by the non-adhesive wedge on a [pacman] pattern as shown in Fig 10e (for movie see S11 Movie). At the left and right side of the [pacman] pattern cells are moving downwards and collide head on at the lower part from where they start to move to the center. The location of the collision fluctuates strongly (compare S11 Movie). The rotation can also occur with reversed direction. To quantify the swirls we calculate the vorticity which is a measure for the rotational tissue flow around a given point. The vorticity has been used before to characterize the impact of cell division on tissue dynamics [84]. A positive vorticity value means counterclockwise rotation of cells in the neighborhood of a central cell. This rotation is observed in the frame of the central cell. Fig 10c shows the vorticity field calculated by Eq 8 for cells on a [circle] pattern. A global counterclockwise rotating swirl of cells is indicated by the large area of positive vorticity at the center of the pattern. The vorticity at the border of the pattern is negative because cells have a lower angular velocity at the border compared to bulk cells. In the frame of bulk cells below the border layer the border cells move backwards resulting in the negative vorticity. The lower angular velocity is a consequence of the finite cell speed. On a circular pattern the outermost cells would need to be the fastest to achieve a constant angular velocity. For the [circular] pattern the cells in the layer below the border already move with the highest speed. Fig 10d shows the vorticity field for the [pacman] pattern. Two swirls rotating in opposite directions are indicated by the opposite signs of the vorticities in the left and right regions.

## Discussion

Our work clearly demonstrates that adhesive micropatterns have a very strong influence on cell dynamics. This agrees well with experimental observations that have motivated our approach. Examples range from the location of daughter cells after cell division [14], cell pairs that are rotating on certain MP but not on others, and MP which guide collective cell migration [28, 33]. By combining the CPM with various model approaches developed earlier for cell mechanics, cell division, cell migration, cell-matrix and cell-cell adhesion, we were able to generate a unifying yet computationally transparent and efficient framework which can explain a large range of experimental observations. This not only contributes to a better understanding of these situations, but in the future will allow us to design adhesive patterns that lead to desired cell dynamics.

The classical CPM [48, 49] describes cells by a phenomenological energy function motivated by the differential adhesion hypothesis (DAH) [50]. It has been extensively used to describe cell ensembles, but has been used only occasionally to describe single cell dynamics [67–70]. Our previous approach to single cells [70] has adjusted the formulation of the energy function for single cells to reflect that they show large differences in adhesive areas on different micropatterns. Mathematically, this is achieved by using an energy function Eq 1 that controls adhesive area by a chemical potential with saturation, rather than by an area-elasticity term that is appropriate when describing cell monolayers with constant thickness. Here we use this approach to first describe how the division plane of cells can be predicted from their shapes. The main idea behind our approach is to map the complex cell shape to a single direction by fitting an ellipse to it at an optimal time point. Fluctuations intrinsic to our modeling approach broaden this single direction into a distribution of directions. The division plane distributions obtained this way agree well with observations for HeLa-cells [14] (compare Fig 3).

Our results allow us to speculate about the underlying mechanisms determining the orientation. During mitosis cells contract to a sphere with an intermediate ellipsoidal shape. During this contraction the mitotic spindle assembles which defines the orientation of the division axis. Our model suggests that the ellipsoidal shape sets the division axis and no direct interaction with the adhesive geometry is required during this stage. Support for this idea comes from the prediction that an ellipsoidal shape is sufficient to align a spindle through its growing astral MT [85] and that the division axis of non-adherent cells is also influenced by their shape [86]. In this view, experiments which speed up spindle assembly compared to cell contraction should result in sharply peaked distributions of the division plane because barely contracted cells are best fit by an ellipse with a high aspect ratio which is less susceptible to fluctuations. We do not model exponential growth in cell volume or projected area before division because we focus on the geometrical and mechanical aspects of cell dynamics and do not include any internal dynamics. In the future, this could be included, similar to the bidirectional coupling between signaling networks and cell shape as modeled before with the CPM [68]. For future studies it would also be interesting to combine division axis prediction and traction force calculation as it has been suggested that traction forces act as memory for the orientation information [87].

For multicellular systems cell-cell adhesion becomes relevant. Many models with an energy based description of cells implement cell-cell adhesion by a decreased line or surface tension at cell-cell interfaces [48, 49, 51, 52, 76]. There are different motivations for this approach. The first one is the DAH developed to explain cell sorting which treats cells similar to molecules in a liquid [50, 76]. Differences in the attraction between cells can lower the energy when cells are rearranged and drives phase separation in this model. The lowered energy can be achieved by a decreased line tension between cells. The second approach is the differential interfacial tension hypothesis (DITH) [88] which explicitly takes into account that cortical tension of individual cells is reduced at cell-cell interfaces [52, 89]. In an energy based description the DAH and DITH cannot be distinguished because they both result in a decrease of the energy when cell-cell interfaces are elongated. However, the response to deformation of an interface in both models is different. For the DAH deforming a cell-medium and cell-cell interface should be resisted in the same way and therefore yield the same line tension because the cortical tension is not changed. For the DITH deformations of the two interfaces should be resisted differently because the cortical tension at interfaces is lower.

There is evidence that adhesion depends on both processes [76]. We therefore describe cell adhesion by a combination of DAH and DITH. At cell-cell interfaces the cortical tension is lowered and we also take the energy required for breaking and forming adherens junctions into account. With this approach we can control adhesion strength and contractility of intercellular interfaces independently. Our CPM can describe cells which require large forces to be separated while their interfaces are strongly contractile with a large tension. Our approach also allows a junction to remain stable for arbitrary adhesion strengths. A pure DITH model could be realized by a reduced fluctuation allowance at interfaces. Indeed actomyosin activity is known to be different at adherens junctions compared to the rest of the cell. Nevertheless this approach would still limit the maximum adhesion strength by the requirement of a positive line tension.

Our approach to cell migration is inspired by the graded radial tension model [77]. Following our focus on cell tension, we addressed cell migration through a reduction of surface tension at the cell front and an increase at the back. This approach does not allow precise control of the underlying actin dynamics [67, 68], however, the shape and dynamic behavior of several cell types are predicted correctly (compare Fig 5c and 5d and S3 Fig). The resulting predictions for the interaction and stability of cell pairs agree well with MCF10A-cells [17]. This requires a lower surface tension as previously reported for MCF10A-cells by means of traction force measurements [70]. A possible explanation is the effect of internal contractile stress fibers which contribute to the overall traction of a cell. These fibers usually do not connect to peripheral stress fibers and therefore do not contribute to the invagination of the contour. In the traction force evaluation [70] their contractile effect was averaged and integrated in the homogeneous surface tension most likely resulting in a higher value as obtained here.

The parameter set we obtain for MCF10A-cells is not unique. The determining factor is the shape of the cells which is described by the dependence of arc radius *R*(*d*) on the spanning distance *d*[70]. Rescaling the shape defining parameters (*σ*, *λ*_*s*_, *k*) and the migratory strength *μ* by the same factor and adjusting the cadherin line energy density accordingly leaves *R*(*d*) unchanged and shape and rotational behavior are the same as before. Only the ratios *k*/*σ*, *λ*_*s*_/*σ* and *μ*/*σ* are relevant for the stability of MCF10A-cells. Absolute values for our parameters can be obtained when our model is compared to traction force measurements. However, the ratios obtained in the current work are roughly 8.5 times larger than in our previous approach for single cells [70]. This indicates that the contributions of the surface tension is reduced for cell pairs compared to single cells. Consistent and unique values for all parameters could be obtained by combining traction force and rotation measurements for cell pairs in a single experiment. Combination with traction force microscopy could also be used to clarify the issue how cell division orientation correlates with shape, stress and strain [74]. In general, it would be very rewarding if one experimental group could measure all our model parameters for one cell type in one set of complementary experiments.

Finally our approach can be applied to collective cell migration. We demonstrated that our CPM correctly predicts the formation of epithelial bridges [28] and swirls [33] (compare Fig 9 and Fig 10). Other multicellular systems that might be studied with this approach are e.g. leader cell formation triggered by curvature [24], closure dynamics above non-adhesive [29] or adhesive gaps [90]. Our implementation is fast enough (typical runtime of several minutes for systems consisting several hundreds of cells) to allow screening of pattern geometries and to address pattern optimization for specific tasks in future projects.

## Models

### The Cellular Potts Model

Our cellular Potts model (CPM) was developed to predict the shape and forces of single cells on MP [70]. Motivated by experimental observations in this context, it pays particular emphasis to the variability of projected cell area and to invaginated shapes over non-adherent regions. Our effective energy function for single non-migrating cells is given by

$$\begin{array}{c} H=\sigma A+\lambda_{s}l+\sum_{\text{arc}\;i}\frac{k}{2L_{0,i}}{\left(L_{i}-L_{0,i}\right)}^{2}-\frac{E_{0}}{A_{\text{ref}}+A_{\text{ad}}}A_{\text{ad}}. \end{array}$$ (1)

The first term results from the surface tension *σ* generated mainly by actomyosin contractility in the cortex. The corresponding energy *σA* scales linearly with the projected cell area *A*. The second term results from the simple line tension *λ*_*s*_ acting throughout the cell contour. The corresponding energy *λ*_*s*_*l* scales linearly with the cell perimeter *l*. The third term accounts for the reinforcement of the cell contour by elastic peripheral actin bundles (tension-elasticity model from [6]). It is only active in concave parts of the cell above non-adhesive areas of the substrate, compare e.g. the free spanning arcs of the cells on the [X] shaped pattern shown in Fig 2a. If the cell has several free spanning arcs, a sum is used in Eq 1. The strength of the arcs is controlled by the elastic arc rigidity *k*. Note however that the way we define it here, it has the physical dimension of a tension. *L*_*i*_ is the contour length of an arc and *L*_*i*, 0_ its rest length. For the rest length we take the spanning distance of an arc which is the length of the straight connection between its endpoints (*d* in Fig 2). The last term in Eq 1 accounts for the gain in adhesive energy. The energy is lowered when the contact area *A*_ad_ with the substrate is increased. The reduction in energy by covering more adhesive area saturates. This reflects the finite amount of adhesion molecules and membrane available to the cell. The ratio of *W* = *E*_0_/*A*_ref_ defines the adhesive energy area density. The value of *A*_ref_ is set through a target cell size *A*_0_ [70]. Note that in contrast to most other formulations of the CPM, we do not use an elastic (harmonic) energy term for the area, because this would constrain it much more than observed during cell adhesion. It is an essential ingredient of our model that cells can transfer material from the third dimension into the adhesive area [71]. Because we do not include an area-elasticity term, surface tension (defined as the derivative of the energy function for surface area) and surface energy area density are identical.

Our implementation of the CPM uses a two-dimensional lattice to represent the cell as illustrated in Fig 2b. All lattice sites belonging to the same cell are marked by the same index. The perimeter of cells is calculated with a refined marching square algorithm [70]. To propagate the cell shapes we use Metropolis dynamics to minimize the cell energy Eq 1. During each step a random lattice site at the cell periphery or the sites surrounding it is selected. The energy

$$\begin{array}{c} \Delta H=H_{\text{new}}-H_{\text{current}} \end{array}$$ (2)

associated with changing it to the values of one of its neighbors is calculated. The step is accepted if Δ*H* < 0 and with the probability exp(−Δ*H*/*T*) otherwise. Here *T* is the fluctuation allowance following from the stochastic nature of the molecular processes underlying changes in cell shape and contractility. Low and high *T*-values correspond to small and large fluctuations, respectively. The time is measured in Monte Carlo sweeps (MCS), where 20.000 MCS correspond roughly to 1 day in real time. During each sweep *n* inversion attempts are performed, where *n* denotes the number of lattice sites in the contour of all cells. For fitting circles to the invaginated arcs, we use a modified least square method [91]. During this fit the endpoints are weighted by a factor of 100 to ensure that the circles run through them.

### Cell Division

We implement cell division by closely following the experimentally observed sequence of events. On the onset of mitosis, stress fibers and focal adhesion disassemble and cells become spherical due to increased myosin contractility [72]. The mitotic spindle assembles and rotates driven by cortical cues. The cortical cues are established through the adhesive geometry prior to division [13]. Thus, the cell divison axis is predetermined during interphase by cell shape [72]. A mapping of the adhesive geometry controlled by MP has been demonstrated to reproduce division axis orientations for many MP geometries [14].

In our approach we put a strong emphasis on the transformation of the flat spread out cell into a sphere. We assume that during this process the spindle assembles and receives cues for its orientation. We let the cell contract and, inspired by the long axis rule [73, 74], fit an ellipse to the cell contour during contraction. The major axis of the ellipse is then taken to be the cell division axis. Increased cortical tension driving a cell to become round during mitosis is achieved in the CPM by increasing the line tension. During rounding the sign of the surface tension is inverted, $\sigma \to \bar{\sigma }=-\sigma$. It takes the role of the osmotic pressure resisting contraction. Setting the simple line tension to $\lambda_{s}\to {\bar{\lambda }}_{s}=-2\sigma \sqrt{A_{b}/\pi }$ ensures contraction, where *A*_b_ denotes the cell area prior to division. Focal adhesion and stress fiber disassembly are achieved by setting the adhesive energy density *W* and elastic arc rigidity *k* to zero. Rescaling the fluctuation allowance by $T\to \bar{T}=T{\bar{\lambda }}_{s}/\lambda_{s}$ ensures the same size of fluctuations before and during roundup. Although fluctuations are expected to decrease during division, this measure is important for the Metropolis algorithm not to get stuck in local minima. The moment of ellipse fitting is defined by the area ratio *r* = *A*/*A*_b_, where *A* denotes the area during contraction. The value of *r* = 0.36 is universal for all patterns. Cell division as simulated with the CPM on an [L] shaped MP is shown in Fig 1. Cell proliferation decreases with increasing cell density and we regulate it through the cell area with a Hill function of the form $r\left(A\right)=r_{0}A^{m}/\left(A^{m}+A_{0}^{m}\right)$ [61]. In this function *r*_0_ denotes the division rate without inhibition by other cells, *A*_0_ the typical cell size, and *m* is the Hill coefficient.

### Cell-Cell Interactions

Cell-cell interactions are mediated by cadherin proteins forming discrete connections between two cells similar to interaction between integrins and the extracellular matrix (ECM) [75]. In contrast to integrin-ECM interactions, however, which are fixed in space, an interface between two cells can deform. Many models [48, 49, 51, 52, 76] reduce the line tension at cell-cell junctions opposed to the cell medium interface based on the argument that surface contacts reduce the apparent intercellular surface tension [92]. This means that the tension acting at cell-cell junctions is set to *λ*_*cc*_ = ∂*H*/∂*l*_*cc*_ = *λ*_*s*_ − *γ*, where *λ*_*s*_ denotes the line tension at a cell-medium interface, *γ* the reduction of it and *l*_*cc*_ the cell-cell interface length. In this way, cells can decrease their energy by increasing the length of cell junctions. An increase of *γ* strengthens the adhesion between cells. The strength of cell-cell adhesion is limited in this approach by the necessity of a positive line tension *λ*_*cc*_. With a very low tension interfaces fluctuate strongly and become unstable or completely dissolve for a negative tensions *λ*_*cc*_. This can be prevented by restricting the cell perimeter to a default length *l*_0_ with a quadratic term (1/2)*κ*(*l* − *l*_0_)^2^ in the energy function [51, 76]. However, the resulting line tension *λ*_*cc*_ = ∂*H*/∂*l*_*cc*_ = *λ*_*s*_ − *γ* + *κ*(*l* − *l*_0_) can still become negative, depends on the global perimeter of the cell and agrees only for *γ*/*λ*_*s*_>1 with experiments [76]. Although cadherins are known to reduce the cortex tension to some extent [52, 89], the tension generated by the cortices of both cells should add up resulting in an higher tension at the junction. Mimicking cell-cell adhesion by a reduced line tension does not allow independent control of cell-cell adhesion strength and contractility of the cell junction.

In our work, we focus on the energy associated with the formation and breaking of discrete cadherin bonds. We picture the cell-cell contact as a zipper where cadherin bonds form and break only where the two parts of the zipper meet. Therefore, the cells energy is only changed when a cell junction is elongated or shortened at the termini of the junction. Elongation by deformation of a junction does not change the energy. As illustrated in the SI S4 Fig a deformation displaces existing cadherin bonds but does not generate new ones. The energy change associated with changes of the cell-cell interface is then

$$\begin{array}{c} \Delta E_{cc}=c\Delta l_{cc}\delta \left(l⇌l_{cc}\right), \end{array}$$ (3)

where *c* denotes the cadherin energy line density, Δ*l*_*cc*_ the change of the cell-cell interface length and $\delta (l⇌l_{cc})$ is one if previously free cell interfaces come in contact to form a cell-cell interface $\left(l⇀l_{cc}\right)$ or break $\left(l↽l_{cc}\right)$. It is zero if *l*_*cc*_ is changed by deformation. A detailed description for a lattice based implementation can be found in S7 Fig. Setting the cell-cell line tension to *λ*_*s*_ effectively means that we attribute a reduced line tension *λ*_*s*_/2 to each of the two contacting cells. This accounts for the fact that cadherins reduce the cortex tension at cell-cell contacts [52, 89]. An illustration of all tensions can be found in Fig 2a.

### Cell Migration

Membrane displacement is driven by actin polymerization at the front and myosin contraction at the back [79]. Actin polymerization and retrograde flow are found to decrease gradually [93] towards the sides of the cell matching the graded radial extension found for keratocytes [77]. With the assumption of a graded filament density and a constant force originating from the membrane tension resisting polymerization, cell shape and motility can be related [94]. In turn, prescribing the cell shape allows to predict the correct actomyosin activities [93]. Coupling both shape and actomyosin can be achieved with a CPM [67, 68]. A reaction-diffusion system for the small G-proteins which drive actin and myosin activity is solved in the domain predicted by the CPM model. Simplified reaction diffusion systems or orientation fields in combination with phase field models also predict correct cell shapes for keratocytes [62, 64] and allow to study cell migration on micropatterns [65].

Because here we aim at a computationally efficient framework, we do not introduce internal fields. Consistent with our tension-based approach, we introduce a graded, direction-dependent tension originating from the polarized actomyosin activity. In its simplest form this tension is given by *μ* cos(*α*) [57], where *μ* is the force per unit length generated at the center of the leading edge and *α* the angle between a point on the membrane and the cell’s polarity direction as depicted in Fig 5a. Actin generated forces at the front (|*α*| < *π*/2) and myosin generated forces at the back (|*α*| > *π*/2) of the cell are equal but with opposite sign. We note that prescribing a force is different from prescribing an extension as in the graded radial extension model [77]. Extension is driven by the resulting force of actin polymerization resisted by membrane tension which depends on the cell shape.

The width of the gradation is known to influence cell shape [93] and can be controlled by taking *μ*|cos(*α*)|^*η*^ sign(cos(*α*)) as actomyosin tension, where sign(*x*) denotes the signum function. The parameter *η* controls the width as shown in Fig 5b. The normal is defined as described in the supporting material of [70] in Fig S3c. In short, a circular mask is applied to each boundary pixel. The normal is defined as the line connecting the center of the circular mask and the center of mass of the occupied pixels in this mask. The radius of the mask is nine pixels. For a typical circular cell with radius of 56 lattice sites (area about 10^4^ lattice sites) the mean absolute error of the theoretical and computed normal orientation is 1.2%.

Protrusions above non-adhesive areas lack the anchoring of adhesions which start to form in the lamella [78]. The polymerizing actin mesh can only reach a certain extension before further polymerization just pushes the mesh backwards. This is put into effect by reducing the protrusion strength by an exponential factor resulting in an effective strength

$$\begin{array}{c} \mu_{\text{eff}}=\mu \;e^{-d/d_{0}}, \end{array}$$ (4)

where *d* is the shortest distance to the nearest adhesive island and *d*_0_ sets the scale on how far protrusions can extend beyond the edge of a MP.

The final energy change associated with cell migration is

$$\Delta H_{m}=-\Delta A{\left|\text{cos}(\alpha )\right|}^{\eta }\text{sign}(\cos(\alpha ))\mu_{\text{eff}}\delta (\text{invasion})]$$ (5)

where Δ*A* denotes the area change. The whole expression is negative because the surface tension is reduced by this amount. The last term accounts for contact inhibition of locomotion. Cells have been demonstrated to coordinate their migration through cell-cell contacts [83]. They do not invade each other actively, which is implemented here by setting *δ*(invasion) to zero if a cell invades another cell. This only happens at the front of the cell (|*α*| < *π*/2). Retraction at the rear is not influenced by contact inhibition.

A commonly used model to describe polarity is a velocity alignment model [45, 65, 80–82, 95]. In this model cells tend to align their polarity with their current velocity. In our implementation of velocity alignment the change of the polarity direction **p** is defined as [80]

$$\begin{array}{c} \dot{p}=v-\frac{p}{\tau }, \end{array}$$ (6)

where **v** is the center of mass (COM) displacement between two Monte Carlo sweeps (MCS) and *τ* sets the time scale of alignment measured in MCS. In Fig 5a the polarity direction **p** points upwards. In general it changes according to Eq 6 after every MCS. The angle *α* used in Eq 5 is always calculated with respect to the current polarity direction. Some models [65, 81, 82] add a explicit noise term to Eq 6. In our approach noise enters implicitly through the finite fluctuation allowance. The COM displacement **v** is not necessarily aligned with the polarity direction due to membrane fluctuations. It certainly gets misaligned when a cell encounters an obstacle. If **v** is treated as a independent function a formal solution for the differential equation Eq 6 can be obtained in the form of

$$\begin{array}{c} p\left(t\right)=\int_{0}^{t}v\left(t^{\prime }\right)\;e^{-\frac{t-t^{\prime }}{\tau }}\;dt^{\prime }, \end{array}$$ (7)

which allows to interpret the mechanism behind velocity alignment. The cell integrates its past displacements with an exponential memory kernel to arrive at its current polarity direction. Cells have been demonstrated to have a memory of the past movement [96].

### Vorticity

We calculate the vorticity *ω* as defined in [84] by

$$\begin{array}{c} \omega =\left(1/A\right)\sum_{r\in O}v_{x}\left(r\right)r_{y}-v_{y}\left(r\right)r_{x}. \end{array}$$ (8)

The region *O* denotes a circular area around a given point, **r** = (**r**_*x*_, **r**_*y*_) is the vector from the center of *O* to the center of a cell in *O* and **v** = (**v**_*x*_, **v**_*y*_) the velocity of this cell. The sum extends over all cells in *O*. *A* is the area of the cell at the center of *O*. This area is used to set the radius of *O* to $R=4\sqrt{A/\pi }$. Hence, the movement of neighboring cells up to second order is used to calculate the vorticity.

### Simulation Parameters

All of our simulation parameters are summarized in Table 1.

## Supporting Information

S1 Fig Orientation of cell division for different aspect ratios of [H] patterns. Probability to find a cell dividing with a certain division axis orientation (= spindle orientation) for different [H] pattern. The aspect ratio *a* is defined as height of the pattern divided by its width. For large aspect ratios the most likely orientation of the division axis is ±90° resulting in daughter cells located at the top an bottom of the pattern. Lowering the aspect ratio shifts the most likely orientation to 0° with the daughter cells located at the left and right side of the pattern. The [H] patterns shown in Fig 3 of the main text have an aspect ratio of 1, 0.91, 0.82 and 0.63. The same parameters as in Fig 3 were used with a bin spacing of 6°.
(EPS)

S2 FigParameter dependence of the division axis.The quality of the predicted spindle orientation is estimated by the norm $L_{2}=\sum_{i}{\left(p_{\text{exp},i}-p_{\text{sim},i}\right)}^{2}/p_{\text{exp},i}^{2}$. The sum extends over the bins in Fig 3 of the main text, *p*_exp,*i*_ denotes the experimental probability to find a division in bin *i* and *p*_sim,*i*_ the respective probabilities predicted by our simulations. The *L*_2_ values are averaged for the 16 patterns investigated. Each map shows how the *L*_2_ value changes as function of a simulation parameter (x-axis) and the area ratio (y-axis). The area ratio sets the moment during contraction when the ellipse is fitted to the cell. It is defined as the quotient of cell area during contraction and the cell area before contraction starts. If not varied, the parameters of a generic cell summarized in Table 1 are used. All maps are capped at *L*_2_ = 0.5 for better visibility. The minimal *L*_2_ value is indicated by a white square. (a) Map as function of the simple tension *λ*_*s*_. The fluctuation allowance is set to *T* = *λ*_*s*_0.06^−1^ to have the same membrane fluctuations for all tensions *λ*_*s*_. The optimal moment to fit the ellipse is independent of the simple line tension *λ*_*s*_. Only for small *λ*_*s*_ (more precisely, small ratios *λ*_*s*_/*σ*) the quality of the predicted distribution changes. Cells are then strongly invaginated and not able to spread across gaps on [L dot], [bar dot] and [two bar] patterns resulting in different initial shapes to predict the division axis. (b) Map as function of surface tension *σ*. The optimal moment to fit the ellipse is independent of the surface tension. As in the previous panel, the quality of the prediction decreases when cells become highly invaginated for large *σ*. (c) Map as function of fluctuation allowance *T*. A higher fluctuation allowance results in larger membrane fluctuations. Information about the cell shape prior to contraction is lost faster and the ellipse needs to be fitted earlier. (d) Map as function of the fraction of remaining adhesive energy density. During contraction the adhesive energy *W*_contract_ remains. Positive values mimic retraction fibers which slow down contraction above adhesive areas. Negative ratios speed up contraction. Optimal agreement is achieved for no remaining adhesion energy during contraction.(EPS)

S3 Fig Trajectory and coupling between cell speed and persistence time. (a) Trajectory of MCF10A-cell on a continuously adhesive substrate. The same parameters as for Fig 6 of the main text were used which result in a persistence length of 40 μm. (b) Dependence of the persistence time on the cell speed. The cell speed was varied by changing the migratory strength *μ* for different gradation widths *η*. The persistence time was calculated as the decay length of angular correlations. Initially, it increases exponentially with the cell speed but saturates for higher speeds as reported for many different cell types [12]. The exponential increase is a result of positive feedback between polarity direction and velocity. In our approach the polarity direction is the sum of past velocities (compare Eq 7 in the main text). If those are random the polarity direction fluctuates resulting again in random movements with random velocities and low persistence time. Increasing the migratory strength *μ* increases the cell speed because membrane extension in polarity direction becomes more likely in the Metropolis dynamics. With the increased speed, fluctuations have less impact on the velocity which stabilizes the polarity direction. In turn, this stabilizes the velocity again leading to a positive feedback and exponential growth. Cell movement can become unstable for very high speeds when cells start to oscillate between right and left turns resulting in a decrease of the persistence time. The MCF10A-cell shown in (a) is moving at a speed of 7μm/10^3^MCS.
(EPS)

S4 Fig Adherens junction formation. New adherens junctions (green) between two cells are only formed when two previously unconnected membrane parts come in contact (top). The yellow part of the cell-cell interface indicates the elongation length *l*_*cc*_ (compare Eq 3 in the main text). Deformation of an interface elongates it as shown in the bottom picture but no new adherens junctions are formed.
(EPS)

S5 Fig Myosin inhibition in rotating cell pairs. Myosin inhibition resulting in a reduction of the surface tension *σ* by a factor of 7 and simple line tension *λ*_*s*_ by a factor of 4.5 as reported in [7] for rat embryonic fibroblasts. The migratory strength *μ* was also reduced by a factor of 4.5. Because of the larger reduction in surface tension cells are less invaginated above nonadhesive areas and have longer cell-cell junctions. The junctions are less contractile and need to be extended less during rotation which makes it easier. For almost all patterns this results in broader distribution meaning more mobile cells. The exceptions are [square] and [C2] which have no or very short concave edges.
(EPS)

S6 Fig Collective cell migration on [comb] pattern with tilted stripes. (a) For the tilted stripes the distance the cells have to bridge increases when they move along the stripes. At some point bridges become to large and movement between the stripes stops. This slows movement along the stripes. Further progress along the stripes is mainly driven by cell division and rearrangement. (b) Comparison of the displacement along the stripes and of bridges for straight stripes as in Fig 9 and tilted stripes as in (a). Progress along the tilted stripes is initially slower compared to straight stripes, but in later phases their velocity is quite similar. Progress along the bridges is initially the same, but when the bridge distance becomes larger the displacement of the bridges for the tilted stripes becomes much slower. (c) Velocities along stripes and bridges. The parameters for HaCaT-cells summarized in Table 1 where used.
(EPS)

S7 Fig Lattice based illustration of the energy associated with the formation of adherens junctions. The lattice sites occupied by two cells labeled A and B are shown. Lattice sites not occupied by a cell have no label. (a) Extension of a cell without the formation of a new cell-cell contact. The cell interface is elongated by two length units indicated by the two red lines along the lattice edges. In the actual implementation the interface length is determined by a refined marching square algorithm. For illustrative purpose the lattice edges are taken here. The energy associated with this interface change is given by Δ*E*_*i*_ = 2*λ*_*s*_, where the factor of two reflects the two length units and *λ*_*s*_ is the line tension associated with cell-medium interfaces. (b) Extension of a cell with the formation of a new cell-cell contact. The energy change is Δ*E*_*i*_ = *λ*_*s*_ + 2*λ*_*cc*_ + *c*, where *λ*_*s*_ accounts for the increase by one length unit of the cell-medium interface. *λ*_*cc*_ is the line tension of each cell at the cell-cell interface. The tensions of both cells add up and result in the factor of two. The energy line density associated with the formation of a new adherens junction is *c*. (c) Extension of a cell by extending an existing contact. The energy change is Δ*E*_*i*_ = 4*λ*_*cc*_. The interface is extended by two length units and the tension of both cells contribute to the energy resulting in a factor of four. For simplicity we choose *λ*_*cc*_ = 0.5*λ*_*s*_ resulting in the same tension at cell-cell and cell-medium interfaces.
(EPS)

S1 Movie Movie of cell spreading and division on [L] shaped micropattern. This movie shows how a single cell spreads out on a [L] shaped micropattern. It then rounds up for division and the two daughter cells spread out again. This movie corresponds to Fig 1.
(MP4)

S2 Movie Cell pair migrating on [square] pattern. This movie shows spreading and migration of a cell on a [square] pattern. The cell then divides and the two daughter cells rotate switching their direction from time to time. This movie corresponds to Fig 6a.
(MP4)

S3 Movie Cell pair migrating on [H] pattern. This movie shows spreading and migration of a cell on a [H] pattern. The single cell is highly mobile exploring the whole pattern. The cell then divides and the two daughter cells are stable and no rotation is observed. This movie corresponds to Fig 6b.
(MP4)

S4 Movie Cell pair migrating on [Reuleaux] triangle pattern. This movie shows a cell spreading and migrating on a [Reuleaux] triangle pattern. It then divides and the two daughter cells rotate with constant speed similar to cells on a circle.
(MP4)

S5 Movie Cell pair migrating on [triangle] pattern. This movie shows a cell spreading and migrating on a [triangle] pattern. It then divides and the two daughter cells do not to rotate persistently. They switch directions frequently.
(MP4)

S6 Movie Collective cell migration into non adhesive regions. This movie shows cells initially confined to a rectangular reservoir. After the confinement is released they migrate into the nonadhesive region forming epithelial bridges. This movie corresponds to Fig 9a.
(MP4)

S7 Movie Collective cell migration into non adhesive regions without bridge formation. This movie shows cells initially confined to a rectangular reservoir. After the confinement is released they migrate towards the nonadhesive region but they cannot form epithelial bridges because the adhesive stripes are too far apart. This movie corresponds to Fig 9b.
(MP4)

S8 Movie Inhibition of cadherins results in retraction of an epithelial bridge. This movie shows an epithelial bridge. After 6s cadherins are inhibited by reducing the cadherin line energy density to half of its original value. The bridge retracts to the reservoir. This movie corresponds to Fig 9c.
(MP4)

S9 Movie Collective cell migration into non adhesive regions of increasing size. This movie shows cells initially confined to a rectangular reservoir. After the confinement is released they migrate towards the nonadhesive and form epithelial bridges. The distance between stripes increases as the cells progress and the bridge formation stops. This movie corresponds to subfigure a of S6 Fig.
(MP4)

S10 Movie Cell migrating collectively forming a swirl on a [circle] pattern. This movie shows cells seeded with a low density on a [circle] pattern. Cells frequently divide and move randomly. As the cell density increases they move coordinated in a single swirl. This movie corresponds to Fig 10a.
(MP4)

S11 Movie Cell migrating collectively forming two swirls on a [pacman] pattern. This movie shows cells seeded with a low density on a [pacman] pattern. Cells frequently divide and move randomly. As the cell density increases two swirls start to form because of the nonadhesive wedge. This movie corresponds to Fig 10b.
(MP4)
